# Multi-Structure-Based Refractive Index Sensor and Its Application in Temperature Sensing

**DOI:** 10.3390/s25020412

**Published:** 2025-01-12

**Authors:** Zhaokun Yan, Shubin Yan, Ziheng Xu, Changxing Chen, Yuhao Cao, Xiaoran Yan, Chong Wang, Taiquan Wu

**Affiliations:** 1 School of Electrical and Control Engineering, North University of China, Taiyuan 030051, China; yzk2321681816@163.com (Z.Y.); chenchangxin@nuc.edu.cn (C.C.); caoyuhao512@163.com (Y.C.); 2 School of Electrical Engineering, Zhejiang University of Water Resources and Electric Power, Hangzhou 310018, China; yanxr@zjweu.edu.cn (X.Y.); wangch@zjweu.edu.cn (C.W.); wutq@zjweu.edu.cn (T.W.); 3 Joint Laboratory of Intelligent Equipment and System for Water Conservancy and Hydropower Safety Monitoring of Zhejiang Province and Belarus, Hangzhou 310018, China; 1240320114@zust.edu.cn; 4 School of Automation and Electrical Engineering, Zhejiang University of Science and Technology, Hangzhou 310023, China

**Keywords:** metal–insulator–metal, Fano resonance, refractive-index nanosensor

## Abstract

In this paper, a new sensor structure is designed, which consists of a metal–insulator–metal (MIM) waveguide and a circular protrusion and a rectangular triangular cavity (CPRTC). The characterization of nanoscale sensors is considered using an approximate numerical method (finite element method). The simulation results show that the sharp asymmetric resonance generated by the interaction between the discrete narrow-band mode and the continuous wideband mode is called Fano resonance. The performance of the sensor is considerably influenced by CPRTC. The sensor structure has attained a sensitivity of 3060 nm/RIU and a figure of merit (FOM) of 53.68. In addition, the application of this structure to temperature sensors is also investigated; its sensitivity is 1.493 nm/°C. The structure also has potential for other nanosensors.

## 1. Introduction

Surface plasmon polaritons (SPPs) are electromagnetic transverse waves that arise when incident light couples with the free electrons at a metal surface. The field strength of this electromagnetic transverse wave decreases exponentially, and its direction is perpendicular to the metal–dielectric boundary [1,2]. The electromagnetic wave is confined to the interface between the metal and the dielectric and does not radiate outwards because the propagation vector of the surface plasmon is larger than that of light in free space. SPPs overcome the conventional optical diffraction limit. Optical information can be transmitted and processed at the nanoscale [3,4]. SPPs also have many good characteristics, such as exceeding the limits of classical light diffraction and controlling the light at the nanoscale. Therefore, SPPs can be applied to a variety of optical devices, such as filters [5,6], optical switches [7,8], logic gates [9], nanosensors [10,11,12,13], and couplers [14,15]. These devices consist of waveguides and resonators and are based on MIM waveguides.

Metal–insulator–metal (MIM) waveguides are one common type of waveguide in SPPs. Compared with traditional optical waveguides, SPP waveguides have a smaller size, higher integration, and stronger optical field localization ability. It is an important part of highly integrated photonic devices in the future [16,17]. Non-linear optical effects such as Fano resonance can also be generated by sensors based on MIM waveguides. An asymmetric line shape characterizes the Fano resonance. The phenomenon was first proposed by Italian–American physicist Hugo Fano while studying inelastic scattering between electrons and helium atoms. At present, the MIM waveguide has been widely studied by many scholars at home and abroad because of its many application prospects. Xiaoyu Zhang et al. [18] proposed a circular ring coupled with an MIM waveguide containing a disc cavity (CRDC), with a sensitivity up to 2240 nm/RIU and an FOM of 62.5. Cheng Zhou et al. [19] proposed an important structure, namely a metal–insulator–metal (MIM) waveguide composed of two stub resonators and a ring resonator, which can be simultaneously utilized as a refractive index sensor and a stopband filter. According to the analysis, by adjusting the aggregation parameters of the MIM waveguide structure, the stopband range and multiple Fano resonance positions can be flexibly and independently tuned. The maximum sensitivity and figure of merit of the MIM waveguide structure are 1650 nm/RIU and 117.8, respectively. Mengmeng Wang et al. [20] proposed a structure in which a circular open-ring resonator cavity is coupled with an MIM waveguide. The structure achieves a maximum sensitivity of 1114.3 nm/RIU and a maximum optimality of 55.71. The two key constraints on the progress of nanosensors in the above research have always been sensitivity and FOM. The sensitivity of the sensor with this structure is 3060 nm/RIU, and the FOM value is 53.68. In comparison, although the FOM value of this sensor has decreased, its sensitivity has seen a significant improvement. Therefore, this sensor plays a notable role when there are high requirements for sensor sensitivity.

The profile of the linear Fano resonance can be altered by modifying the dimensions of the construct, thereby increasing the efficiency of the device. Generally, Fano resonance is regarded as a unique characteristic resonance of quantum systems, which is generated by the interference between narrow discrete states and wide continuous states. The interaction between two or more oscillatory or resonant modes with very close frequencies can produce interference phenomena. Specifically, interference between a wide line width with resonance and a narrow line width can result in a resonance with a significantly asymmetric line width. Sharp asymmetric linear Fano resonances have a relatively small full width at half maximum (FWHM). The FWHM can be used as an indicator of device performance: the tighter the FWHM, the better the device performance.

In this paper, innovating on the original cavity structure, an MIM device with a CPRTC structure is presented. To further demonstrate the standardized Hz field distribution and propagation characteristics of this new structure, a rigorous and detailed analysis is carried out using the finite element method (FEM). Given that the spectrum of the Fano resonance is very sensitive to the structural values of the system, several key parameters including the outer radius of the CPTRC, the radius of the two protrusive circles, the angle at which the cavity rotates to different positions, and the coupling distance were investigated in depth. In addition, a further discussion of the sensor’s wide range of applications in temperature measurement was carried out, which provides a new idea and method for a temperature detection field.

## 2. Geometric Model and Analysis Method

**Figure 1:**
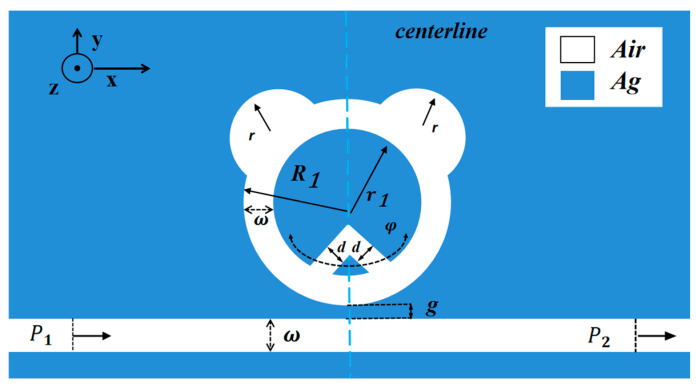
Two-dimensional structure diagram.

Since the epidermal depth of the SPPs is very shallow and much smaller than the height of the designed structure, for convenience and intuition, we used a 2D model for the calculations. Figure 1 shows the two-dimensional representation of the designed structure. The whole sensor structure is composed of a CPRTC structure and a waveguide in centerline symmetry. R1 is the outer radius of the ring, r1 is the inner radius of the ring, the ring width is equal to the width of the MIM waveguide, both are expressed by ω, d is the distance between the rectangular triangle, r is the circular projection radius, and g signifies the coupled spacing between the waveguide and CPRTC structure. To ensure that the pattern of electromagnetic waves is horizontal, ω is 50 nm in this paper. In this study, the value of d is also 50 nm. The dark part is silver medium, and the white part is air.

Since silver has a lower power consumption, that is, the dielectric constant of silver is high at lower frequencies (especially well below the plasma frequency), its permittivity can be defined by the Debye–Drude dispersion model:

(1) $$\varepsilon \left(\omega \right)=\varepsilon_{\infty }+\frac{\varepsilon_{s}-\varepsilon_{\infty }}{1+i\tau \omega }+\frac{\delta }{i\omega \varepsilon_{0}}$$

where $\varepsilon_{\infty }=3.8344$ is the infinite frequency relative permittivity, $\varepsilon_{s}=-9530.5$ is the static dielectric constant, the relax time is $\tau =7.35\;*\;10^{-15}\;s$, and the permittivity of the silver is $\delta =1.1486*10^{-7}\;s/m$ [21]. The transverse magnetic mode (TM modal) equation is [22,23]

(2) $$\tanh\left(k\omega \right)=-\frac{2k\alpha_{c}}{k^{2}+p^{2}\alpha_{c}}$$

In the formula, the wave vector is represented by k, p represents the relative permittivity ratio, expressed as $p=\varepsilon_{in}/\varepsilon_{m},{\;\alpha }_{c}$ is the attenuation constant, which describes the degree of amplitude attenuation of the electromagnetic wave during its propagation in the waveguide due to various factors (such as conductor loss, dielectric loss, etc.). It is expressed as ${\;\alpha }_{c}={\left[{k_{0}}^{2}*(\varepsilon_{in}-\varepsilon_{m})+k\right]}^{\frac{1}{2}};\varepsilon_{m}$ and $\varepsilon_{in}$ represent the values of the permittivity of the metal and the dielectric, respectively. When the medium is air, k can be expressed as $k_{0}=2\pi /\lambda_{0}$.

Using the two values of sensitivity and FOM allows for a better inference of the effectiveness of the sensor, and their calculation formula is as follows [24]:

(3) S=Δλ/Δn

(4) FOM=S/FWHM

where ∆λ and ∆n represent the resonance wavelength change and the index of refraction change, respectively. S represents sensitivity. FWHM represents the full wave at half maximum, with a value of 57.0 in the text.

We use powerful COMSOL Multiphysics 5.4a software to simulate the transmission spectrum of this structure. Considering that nanoscale devices face many difficulties in the actual manufacturing process, in order to reduce the production cost, we can improve the performance of the sensor by improving its parameters. In view of this, we carefully choose the hyperfine mesh partitioning method to maximize the precision of the computational results. In terms of the choice of light source, because ordinary light is usually difficult to meet the specific conditions of the structure simulation, after in-depth consideration and comparison, we finally decided to use laser as a light source. This is because the laser has extremely good monochromatic difference and correlation, which can provide more stable and reliable lighting conditions for our simulations. In addition, the boundary conditions are implemented as fully matched layers at the top and bottom of the structure. These can effectively dampen overflow waves, ensuring a reasonable distribution of energy and the accuracy of the calculation during the simulation.

## 3. Simulation and Results

**Figure 2:**
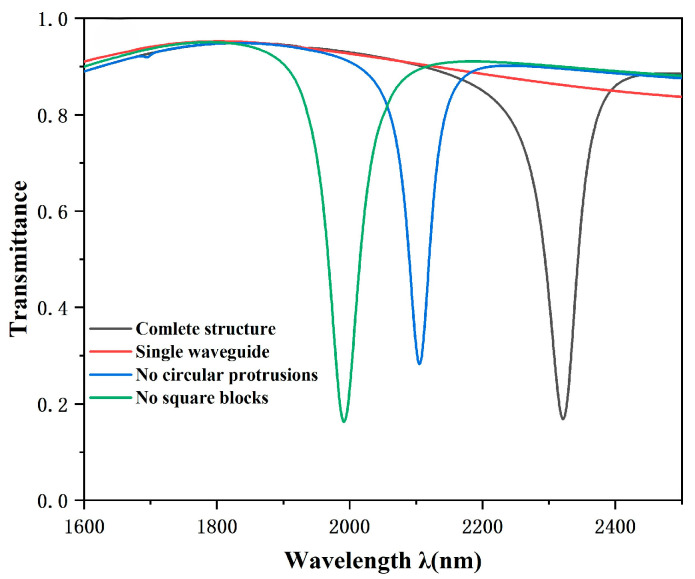
The transmission curve of a structure with a complete CPRTC is shown as a black curve, the curve of a structure without a circular protrusion is shown as a blue curve, the structure without rectangular triangles is shown as a green curve, and the structure with a single waveguide is shown as a red curve. The arrows indicate the distribution of the magnetic field in the corresponding wave valley.

In order to fully demonstrate the significant advantages of the created structures in terms of transmission characteristics, we carry out a thorough and detailed comparative analysis. In particular, we make a comprehensive comparison of the whole system, a single waveguide, non-circular protrusions, and non-rectangular triangular cavities. The projected spectrum is shown in Figure 2, where four different coloured lines clearly represent a single waveguide, complete structure, no circular protrusions, and no rectangular triangular cavity, respectively. These structures all have the same parameter settings; the outer ring radius R1 is 240 nm, the circular projection radius r is 120 nm, the rectangular triangle cavity radius d is 50 nm, and the coupling distance g is 15 nm. After in-depth study, it is found that the single MIM waveguide represented by red has a light transmittance that is always stable in the range of 0.8 to 0.95. In this interval, the transmittance is always at a high level, and its projected spectral map almost shows a horizontal line; because of this, we can regard it as a broadband mode. However, the other three curves, namely the black, green, and blue curves, have different valley bottoms, relatively low light transmittance, and extremely narrow spectral widths. Based on these characteristics, we can consider these three structures as narrow-band patterns. In addition, the three curves all show an asymmetric shape, which fully indicates that the three structures have successfully caused Fano resonance.

To better comprehend the outstanding benefits of the CPTRC structure, we have performed a detailed comparative analysis of the green and black curves. It can be seen that the overall curve under the full structural design has shifted significantly to the right compared to the previous curve. This significant shift means that the accuracy of the sensor has been greatly enhanced. We then observed the magnetic field and found that the strength of the magnetic field had weakened slightly, and this phenomenon was entirely consistent with the slight increase in light transmission. Then, we compare the two curves of blue and black, from which we can find that the structure without the circular protrusion shows a relatively weak magnetic field, but the transmission rate is higher, and the position of the valley bottom is also higher. Moreover, the semi-bandwidth of the structure without circular protrusions is large, which directly leads to the decline of the system’s optimal value. By comparing different curves and observing parameters such as magnetic field and transmittance, we can understand the characteristics and advantages of the CPTRC structure more comprehensively and also provide a valuable reference direction for further optimization and improvement of the structure.

**Figure 3:**
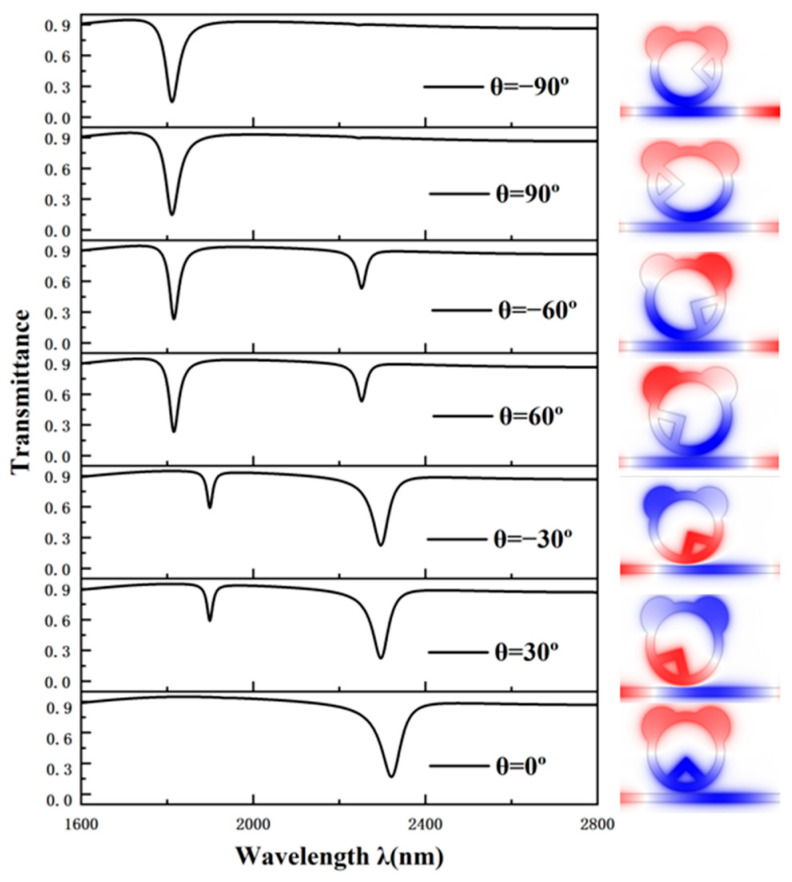
The projected spectra and electric fields of rectangular triangular cavities with different angles.

If the rectangular triangular cavity is in different positions on the ring, it will produce different propagation characteristics. Therefore, it is stipulated that φ is positive when rotating clockwise, and the system performance is compared when φ is 0°, 30°, −30°, 60°, −60°, 90°, and −90°. Its projected spectra are shown in Figure 3. When clockwise and counterclockwise rotation are at the same angle, the spectra produced are similar. Therefore, we can only study the spectral diagram of φ > 0°. When φ = 0°, there is a dip angle with low transmittance and a large wavelength asymmetry curve, indicating that this structure has a high sensitivity. When φ = 30°, there are two dip angles, a high transmittance, and a low projection rate; the lower transmittance corresponding to the dip wavelength is also relatively large, but compared with φ = 0°, transmittance is high, resulting in its corresponding optimal value becoming low. When φ = 60°, it also has a high and a low transmittance dip angle, but the lower transmittance dip angle corresponds to a lower wavelength, indicating that the structure is less sensitive. When φ = 90°, it has a low transmittance dip angle and a smaller wavelength asymmetry curve, indicating that the sensitivity of this structure is low. The above results show that the CPRTC has the best performance when φ = 0°.

Then, to further investigate the impact of the index of refraction on system performance, we carefully set six different refractive index parameters, which are 1.00, 1.01, 1.02, 1.03, 1.04, and 1.05, and we carried out detailed experiments.

**Figure 4:**
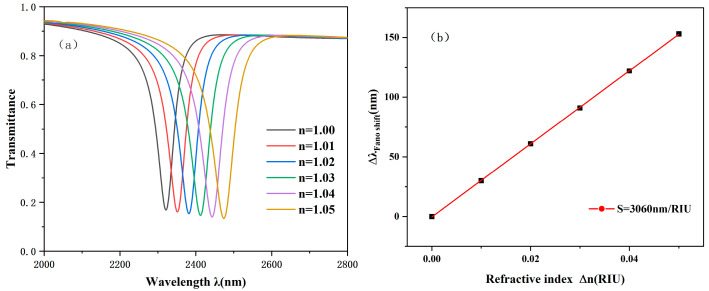
(**a**) Projection spectrogram at different refractive indices. (**b**) Sensitivity fit lines at different n values.

The results of the simulation are illustrated in Figure 4, clearly showing the performance of the system under different refractive indices. It can be clearly seen that the refractive index n gradually increases by the same value as it changes and an approximately equal redshift phenomenon will also occur on the spectrum, as shown in Figure 4a. Through the in-depth analysis and calculation of the experimental results, we successfully achieved the maximum sensitivity of 3060 nm/RIU and a high FOM of 53.68.

**Figure 5:**
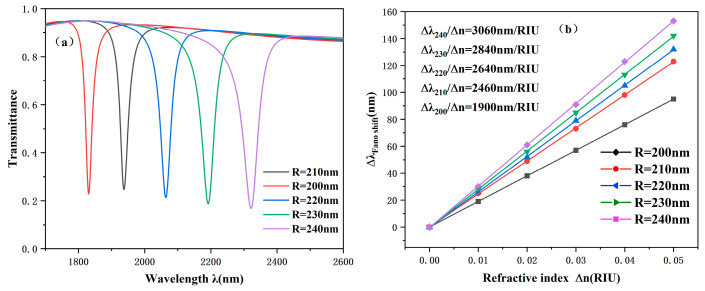
(**a**) Projected spectrum when the outer radius of the circle is changed. (**b**) Sensitivity fitted lines for different values of the outer radius of the rings.

The effect of R1 transformation on the performance of CPRTC is studied in detail. Specifically, we set R1 to be 200 nm, 210 nm, 220 nm, 230 nm, and 240 nm, respectively, to carry out the experiment. The experimental results are shown in Figure 5, which clearly shows the performance of the system under different R1 values. With the increase in R1, the spectrum also has an obvious redshift to the right. This is because increasing R1 increases CPRTC’s effective length. However, the changes in light transmittance and FWHM were not significant. According to Figure 5b, we can learn that the sensitivity changes from 1900 nm/RIU to 3060 nm/RIU as R1 increases. This fully shows that R1 is a crucial parameter in structural parameter changes. In practical applications, we can carefully select the appropriate size according to the specific sensitivity requirements of the equipment manufacturing involved. In this way, the performance requirements of the device can be met and resources can be properly used and optimized.

**Figure 6:**
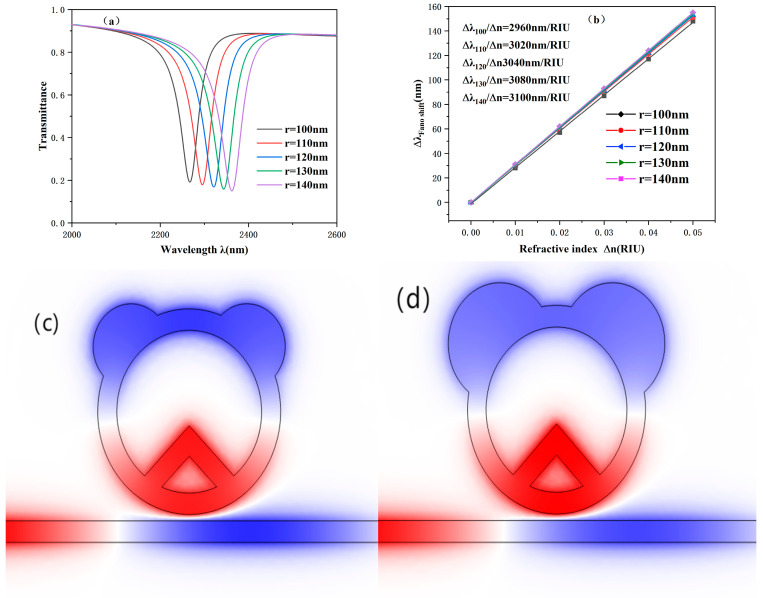
(**a**) Projected spectra at different values of circular protrusion radius. (**b**) Sensitivity fit lines for different values of circular protrusion radius. (**c**) Electric field map at a radius of 100 nm. (**d**) Electric field map at a radius of 140 nm.

Furthermore, the effect of changes in r on the system is also part of our discussion; we adjusted the value of r, gradually changing it from 100 nm to 140 nm. According to Figure 6, the transmission spectra of the different r-generated structures and the electric field distributions of the two structures at r of 100 nm and 140 nm, respectively, can be clearly seen. As r continues to increase, it can be observed that the change in transmittance is not obvious. However, the projected spectrum has a very obvious redshift phenomenon. At the same time, compared with other regions, the field strength inside the ring cavity is significantly larger. We can see that as r grows, so does the effective area of the circular projection. That is, the ability of a larger circular protruding cavity to gather a stronger electric field on the ring becomes stronger. As shown in Figure 6b, the sensitivity shows a strong linear correlation. However, as r increases, there is no obvious change in the sensitivity fitting line, and there is no significant increase in the system sensitivity. It can be seen that changing r can improve sensitivity in a small range. In practical application, we can adjust the value of r reasonably according to the specific needs to optimize the behaviour of the system to a limited extent.

**Figure 7:**
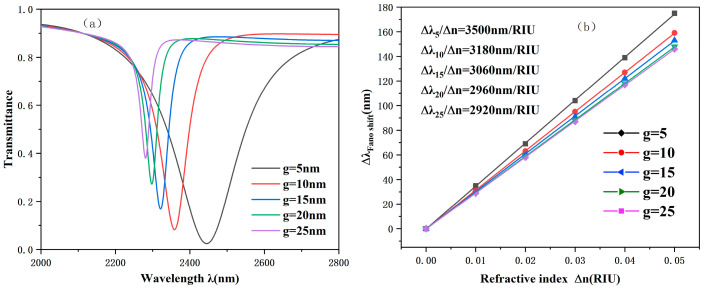
(**a**) Projection spectrum at different g values. (**b**) Sensitivity fitting lines at different g values.

Finally, the effect of the distance g of the coupling on the performance of the system is studied. The value of g in the range of 5 to 25 nm was changed to evaluate the specific impact of the coupling gap on system performance. As shown in Figure 7, with the increase in the g value, the FWHM gradually narrowed, the lowest point of the image rises, and the transmission increased accordingly. This phenomenon fully indicates that the coupling between SPPs and CPRTC structures becomes more and more difficult. As shown in Figure 7b, when g = 5, the system sensitivity can reach 3500. However, at this time, the transmission is too large, which makes the coupling of SPPs and the structure face great difficulties. When g is 10 nm, 15 nm, 20 nm, and 25 nm, the change in system sensitivity is not obvious. Therefore, in practical applications, we should choose a suitable g value to obtain a low transmittance spectrum. At the same time, the FWHM should not be too large, because a too large FWHM will cause the system value to drop sharply and then seriously damage the performance of the system. When g > 15, the transmittance becomes larger, which means that coupling the SPPs to the CPRTC structure does become more difficult. When g < 15, the FWHM will increase sharply, resulting in a sharp decline in the optimal value. Taking various factors into consideration, finally, a coupling distance of 15 nm is set to make the sensor work best. Such a choice can not only ensure the sensitivity of the system to a certain extent but also avoid adverse effects on the system performance due to excessive transmission or FWHM.

Finally, we drew a comparison diagram of sensor characteristics between this structure and other structures, as shown in Table 1.

## 4. Structural Applications

The sensor studied has a number of significant advantages and its simple structure has a very high sensitivity; because of this, it can be used as a temperature sensor. In practice, this structure is very easy to miniaturize and integrate. The fabrication process is also uncomplicated and can be manufactured by simply etching a silver deposition film with a thickness of 100 nm on a quartz substrate. Compared to silver and quartz, ethanol’s refractive index is two orders of magnitude greater. When ethanol is filled into the CPRTC structure, the error caused by the ambient temperature change is negligible. Therefore, filling the bus waveguide and CPRTC with ethanol to act as the sensitive medium is a very suitable choice. In addition, methanol, propylene glycol, etc., can all serve as sensitive media. Ethanol has a boiling point of 78 °C and a melting point of −144 °C. In this temperature range, the refractive index of ethanol will change linearly with the increase or decrease in temperature, which can be linearly fitted by the following Formula (5):

(5) $$n=1.36048-3.94*10^{-4}(T-T_{0})$$

where T represents the temperature of the environment being tested and T0 represents room temperature, which is specified here as 20 °C, considering that ethanol is liquid only between −144 °C and 78 °C. the operating range of this sensor should be between 78 °C and −144 °C. The geometric parameters are set with sensitivity consistent with the structural parameters at the optimum of the system. The temperature is set to −80, −45, −10, 25, and 60 °C. Since changes in ambient temperature change the refractive index of ethanol, temperature sensors convert the detected displacement into information about the temperature change through the phenomenon of spectral displacement.

**Figure 8:**
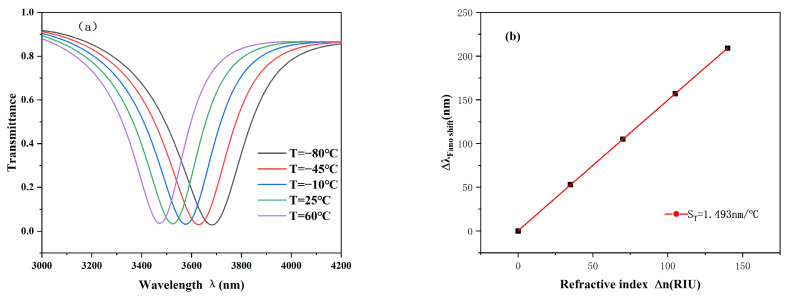
(**a**) Transmission spectrum at a specified temperature. (**b**) Sensitivity fit curve at a specified temperature value.

The S value of the temperature sensor can be calculated by an analogy with the formula for calculating the sensitivity of the refractive index sensor (Formula (3)), where ∆λ represents the projected spectral shift and ∆T represents the temperature change. In our experiments, we designed a temperature range of −80–60 °C; the refractive index variation range of the structure designed can be computed from Formula (5) as 1.39988~1.34472. Figure 8 shows that as the temperature increases, the projected spectrum also moves to the left. The lowest point of the image corresponds to a shift in wavelength from 3473 nm to 3682 nm, which means a delta of 209. Figure 8b fully illustrates that the temperature change and the projected spectral displacement have a good linear fit. After detailed calculation, the sensitivity of the temperature sensor is 1.493 nm/°C. This level of sensitivity is of great value in practical applications and can provide more accurate results for temperature measurement. At the same time, the structural characteristics and working principle of the sensor also provide a new idea and method for the development of a temperature-sensing field.

## 5. Conclusions

In this study, we propose a novel and accessible nanoscale sensor structure for temperature detection, which consists of am MIM waveguide and CPRTC. This structure is relatively simple and relatively easy to fabricate. The results show that the narrow mode determined by the CPRTC structure interferes with the broadband mode determined by the waveguide, and the Fano effect appears in the general transmission. The external radius R1 and the circular protrusion cavity have great influence on the system performance. By adjusting the structural parameters of the system, the performance of the sensor is greatly improved. The structure has a maximum sensitivity of 3060 nm/RIU and an FOM of 53.68. The sensor of the structure also has good sensitivity for temperature detection, which can reach a sensitivity of 1.493 nm/°C. Therefore, it has a promising application prospect in the manufacturing process of semiconductor chips, where extremely high precision in temperature control is required. Nanoscale temperature sensors can be installed on tiny components of key equipment such as lithography machines and etching machines to monitor temperature changes in real time. This enables a timely adjustment of process parameters, ensuring the quality and stability of chip manufacturing and improving production efficiency and the yield rate of qualified products. This sensor can also be applied to detect alcohol concentration and sodium ion concentration in blood, thereby serving in the medical field. The designed structure could also potentially be applied to other plasma optics in the future.

## Figures and Tables

**Table 1:** Results are compared with recent studies.

References	Operating Wavelength Range	Sensitivity (nm/RIU)	FOM
[25]	1300 nm < λ < 1900 nm	1100	224
[26]	800 nm < λ < 1600 nm	1200	126
[27]	1000 nm < λ < 1600 nm	1420	76.76
This work	1700 nm < λ <2500 nm	3060	53.68

## Data Availability

The available data have been stated in the article.
